# Comparison of intestinal microbes in female and male Chinese concave‐eared frogs (*Odorrana tormota*) and effect of nematode infection on gut bacterial communities

**DOI:** 10.1002/mbo3.749

**Published:** 2018-10-11

**Authors:** Yilin Shu, Pei Hong, Dong Tang, Hui Qing, Oscar Omondi Donde, Huan Wang, Bangding Xiao, Hailong Wu

**Affiliations:** ^1^ Key Laboratory for the Conservation and Utilization of Important Biological Resources of Anhui Province Wuhu China; ^2^ College of Life Sciences Anhui Normal University Wuhu China; ^3^ Key Laboratory of Algal Biology of the Chinese Academy of Sciences, Institute of Hydrobiology Chinese Academy of Sciences Wuhan China; ^4^ University of Chinese Academy of Sciences Beijing China; ^5^ Department of Environmental Science Egerton University Egerton Kenya

**Keywords:** intestinal microorganism, metagenome, nematode infection, *Odorrana tormota*, sexual dimorphism

## Abstract

The Chinese concave‐eared frog (*Odorrana tormota*) is a rare and threatened species with remarkable sexual dimorphism. Intestinal microbes are understood to play important roles in animal physiology, growth, ecology, and evolution. However, little is known about the intestinal microbes in female and male frogs, as well as the contributing effect by gut infesting nematodes to the co‐habiting bacteria and their function in degradation food rich in chitin. Here, this study analyzed the microbiota of the intestinal tract of both female and male, healthy as well as nematode‐infested concave‐eared frogs using high throughput 16S rRNA sequencing and metagenomic techniques. The results showed that the bacterial composition of the microbiota at the phylum level was dominated by Firmicutes, Verrucomicrobia, Bacteroidetes, and Proteobacteria. The study also revealed that the community composition below the class level could be represent sex differences, particularly with regard to Enterobacteriales, Enterobacteriaceae, Peptostreptococcaceae, and Rikenellaceae, among others. Carbohydrate‐active enzyme‐encoding genes and modules were identified in related gut bacteria by metagenomic analysis, with Bacteroidia, Clostridia, and gammaproteobacteria predicted to be the main classes of chitin‐decomposing bacteria in the frog intestine. In addition, the abundance of some bacteria significantly increased or decreased in nematode‐infected hosts compared with healthy individuals, including Verrucomicrobia, Verrucomicrobiae, Negativicutes, Actinobacteria, and Bacilli, among others. This indicates that nematode infection may affect the richness and composition of some gut bacteria.

## 1 INTRODUCTION

The gastrointestinal tract is the primary site where microorganisms interact with the host species. The intestinal microbiota can develop a natural defense barrier exerting different protective, structural, and metabolic effects on the host epithelium (Gaskins, Croix, Nakamura, & Nava, 2008; Ivanov & Littman, 2011). The diversity of the frog gut microbiota was influenced by hibernation, metamorphosis, environmental pollution, and other factors (Van der, Cohen, & Nace, 1974; Jennifer, Loesche, & Nace, 1982; Kohl, Cary, Karasov, & Dearing, 2015). At present, research on intestinal microbes has generally been limited to a few frog species (Huang, Chang, Huang, Gao, & Liao, 2018; Kohl, Cary, Karasov, & Dearing, 2013; Vences et al., 2016; Wiebler, Kohl, Lee, & Costanzo, 2018).

Diet, as an important environmental factor, serves as both a source of bacteria and a change in the nutritional environment of the intestines (Costello, Stagaman, Dethlefsen, & Bohannan, 2012; David et al., 2014; Janssen & Kersten, 2015; Vences et al., 2016). Different diets can vary in their macronutrient content and therefore they might favor certain bacterial communities of the host (Knutie et al., 2017). For amphibian groups, most species show sexual dimorphism, with females larger than males (Shine, 1979).The males are likely to be limited by the size of the body as well as to the feeding organs, making it impossible to hunt larger volumes of food (Houston, 1973; Toft, 1980). Indeed, according to the theory of optimal foraging, larger frogs tend to prey on larger rather than smaller foods (Hirai, 2002; Lima & Moreira, 1993). Sex difference also affects the intestinal microbial composition (Costello et al., 2012; Freire, Basit, Choudhary, & Chee, 2011; Koren et al., 2012; Kovacs et al., 2011; Markle et al., 2014). To date, the effect of sex on the gut microbiota of amphibians has not been adequately explored.

The Chinese concave‐eared frog (*Odorrana tormota*) is the first non‐mammalian vertebrate shown to be able to communicate using ultrasound (Feng et al., 2006). It is only found in eastern China, mainly in the southern mountains of Anhui Province and the western mountains of Zhejiang Province (Fei, 1999; Feng, Zhang, Shu, & Yao, 2015). Because of its limited and fragmented distribution, the wild population is classified as a vulnerable species by the International Union for the Conservation of Nature and Natural Resources. *O. tormota* demonstrates sexual dimorphism, with females being significantly larger than males. Males have an average snout‐to‐vent length (SVL) of 32.5 mm, whereas females average 56 mm (Feng, Narins, & Xu, 2002). Adult frogs mainly feed on insects, including Lepidoptera, Arachnida, Hymenoptera, and Orthoptera species, as well as damselflies (Fei, 1999). The ratio of intestine length to SVL varies between 0.44 and 0.91, which is the lowest known ratio among the Anura (Wu, Xiong, Lei, & Jiang, 2012). Therefore, how the concave‐eared frog obtains enough energy from hydrolyzing chitin, the major component of the insect shell, in such a short gut needs to be further examined, as does the role of gut microbes in this process. Additionally, very little is known about the effects of pathogens, such as intestinal parasites, on the gut microbiota of most frogs.

Therefore, this study was aimed at comparing the gut bacterial communities between male and female Chinese concave‐eared frogs using a 16S rRNA‐based sequencing method. Additionally, metagenomic analysis was used to explore the potential function of the gut bacteria, especially the role of the gut bacteria in the biodegradation of chitin by frogs. Furthermore, the microbial communities of healthy and nematode‐infected individuals were compared with the aim of evaluating the effects of intestinal parasites on the gut microbial communities of frogs.

## 2 MATERIALS AND METHODS

### 2.1 Experimental animals and sample collection

Fifteen concave‐eared frogs, including seven females and eight males, were collected from Banqiao Provincial Natural Reserve, Anhui Province, China, during the 2017 breeding season. All individuals were separately placed into plastic boxes containing plant leaves and water from their natural environment and transported to the laboratory for further analyses. After being starved for 3 days, intestinal contents were collected from the midgut and small intestines as described in Mashoof, Goodroe, Du, and Eubanks (2013). All samples were then stored at −80°C until further processing. Among the 15 frogs, four (one female and three males) were found to be nematode‐infected after dissection.

To assess the effects of sex on the gut microbiota, five male individuals (RTM1–RTM5) and six female individuals (RTF1–RTF6) were compared. To assess the effects of nematode infection on the gut microbiota while controlling for the influence of sex, all three infected male individuals (Infect2–Infect4) and the five normal male individuals (RTM1–RTM5) were separated into infected and uninfected groups for further study. All frogs were determined to be 2 years of age on the basis of skeletochronology (Tsiora & Kyriakopoulou‐Sklavounou, 2002; Supporting Information Figure S1).

### 2.2 DNA extraction

A FastDNA SPIN Kit for soil (MoBio Laboratories, Carlsbad, CA) was used to extract DNA from the samples according to the manufacturer’s instructions. DNA quality was examined by 1% agarose gel electrophoresis and measured by spectrophotometry. All DNA samples were stored at −20°C until further processing.

### 2.3 16S rRNA gene amplification, sequencing, and processing of sequencing data

16S ribosomal RNA (rRNA) genes were amplified from all samples for barcode‐based sequencing. We amplified the V3–V4 region of the bacterial 16S rRNA gene using the universal forward primer 338F (5′‐ACTCCTACGGGAGGCAGCAG‐3′) and the reverse primer 806R (5′‐GGACTACHVGGGTWTCTAAT‐3′; Xu, Wang, Gai, & Xia, 2016). Purified amplicons were pooled in equimolar concentrations and paired‐end sequenced (2 × 300) on an Illumina MiSeq platform (Illumina, San Diego) by Majorbio Bio‐Pharm Technology Co. Ltd. (Shanghai, China) according to standard protocols.

Operational taxonomic units (OTUs) were clustered with a 97% similarity cutoff using Usearch (version 7.0 https://drive5.com/uparse/) and chimeric sequences were identified and removed using UCHIME. The taxonomy of each 16S rRNA gene sequence was analyzed using the RDP Classifier algorithm (https://rdp.cme.msu.edu/) against the Silva (SSU123) 16S rRNA database using a confidence threshold of 70%.

### 2.4 Metagenomic sequencing, quality control, and genome assembly

To characterize and compare the microbial communities in the intestines of the male and female concave‐eared frogs, two metagenomic DNA samples were sequenced. For the male sample, equal quantities of total DNA were isolated from five individual frogs and pooled, while for the female sample, equal quantities of total DNA were isolated from six individual frogs and pooled. DNA was fragmented to an average size of about 300 bp for paired‐end library construction using a Covaris M220 ultrasonicator. Paired‐end libraries were prepared using a TruSeq DNA Sample Prep Kit (Illumina). Adapters containing the full complement of sequencing primer hybridization sites were ligated to the blunt‐end fragments. Paired‐end sequencing was performed on a HiSeq4000 platform (Illumina) at Majorbio Bio‐Pharm Technology using a HiSeq 3000/4000 PE Cluster Kit and HiSeq 3000/4000 SBS Kits according to the manufacturer’s instructions. Each read was then trimmed using Sickle (https://github.com/najoshi/scickle). Reads that aligned with the *Xenopus tropicalis* and *Nanorana parkeri* genomes, as determined by BWA (https://bio-bwa.sourceforge.net), and any hits associated with the reads and their mated reads were removed. The resultant high‐quality reads were then used for further analysis. The Illumina reads were assembled into contigs using IDBA‐UD (Peng, Leung, Yiu, & Chin, 2012) with default parameters.

### 2.5 Gene prediction, taxonomy, and functional annotation

Genes were predicted within the contigs using MetaGeneMark (Zhu, Lomsadze, & Borodovsky, 2010). A non‐redundant gene catalog was constructed with CD‐HIT (Li & Godzik, 2006) using a sequence identity cutoff of 0.95, with a minimum coverage cutoff of 0.9 for the shorter sequences. This catalog contained 982,379 microbial genes (Supporting Information Table S1). Gene reads were characterized using BLASTX (Altschul, Madden, Schäffer, & Zhang, 1997) comparisons against the integrated NCBI non‐redundant (nr) protein database (E‐values <10^−5^). The LCA‐based algorithm implemented in MEGAN (Huson, Auch, Qi, & Schuster, 2007) was used to determine the taxonomic level of each gene. MetaGene Annotator (Noguchi, Park, & Takagi, 2006) was applied to the assembled contigs to identify open reading frames (ORFs) longer than 100 bp. ORFs were translated using the Bacterial Genetic Code. BLASTP (Version 2.2.28+, https://blast.ncbi.nlm.nih.gov/Blast.cgi) was used for taxonomic annotations by aligning non‐redundant gene catalogs against the NCBI nr database with an e‐value cutoff of 1e^−5^. Clusters of orthologous groups (COG) analysis of the annotated ORFs was performed using BLASTP against the eggNOG database (v4.5) with an e‐value cutoff of 1e^−5^ (Jensen et al., 2008; Tatusov, Fedorova, Jackson, & Jacobs, 2003). Carbohydrate‐active enzymes were annotated using hmmscan (https://hmmer.janelia.org/search/hmmscan) against the CAZy database (v5.0; https://www.cazy.org/) with an e‐value cutoff of 1e^−5^ (Rice, Longden, & Bleasby, 2000).

### 2.6 Statistical analysis

Several α‐diversity measurements were calculated for each sample. The Shannon index, Simpson’s index, and the Good’s coverage index were calculated to estimate diversity. Chao1 was also calculated to estimate OTU richness. All diversity metrics were then compared using the Mann–Whitney *U* test.

To identify taxa with different abundance between healthy and nematode‐infected frogs, the LDA Effect Size (LEfSe) algorithm was used through an online Galaxy interface (https://huttenhower.sph.harvard.edu/galaxy/root). This performed non‐parametric factorial Kruskal–Wallis sum‐rank tests and linear discriminant analysis (LDA) to determine whether these features are consistent with the expected behavior of the different biological classes (Segata et al., 2011).

To compare community compositions between groups, analysis of similarities (ANOSIM) and non‐metric multidimensional scaling (NMDS) was conducted to investigate dissimilarities between healthy and nematode‐infected individuals. ANOSIM was conducted using a Bray–Curtis index of similarity with 9999 permutations. *R* values indicate the biological importance of differences, ranging between −1 and 1. The closer *R* was to 1, the greater the difference between groups than within groups. NMDS analysis was performed in the R “vegan” package (Oksanen, Kindt, Legendre, & Hara, 2007) using a Bray–Curtis index.

## 3 RESULTS

### 3.1 Concave‐eared frog dataset

Overall, the dataset consisted of 546,643 high‐quality 16S rRNA gene sequences, with an average of 439 sequences for each of the 15 samples (Supporting Information Table S2). OTUs were delineated at a 97% similarity level, leaving 406,905 sequences for further analysis (Supporting Information Table S3). The *p*‐value of <0. 05 indicated that the difference between groups was significantly larger than that within groups.

### 3.2 Gut microbiota of female vs. male concave‐eared frogs

**Figure 1:**
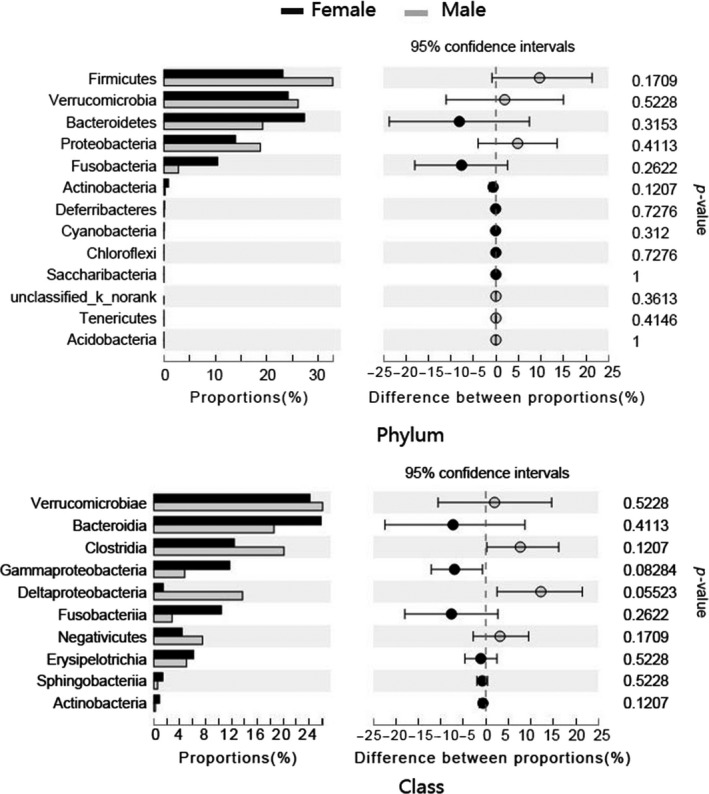
Comparison of the taxonomic compositions of the gut microbiota of male and female Chinese concave‐eared frogs. Relative abundances (percentage) of the microbiota at the phylum and class levels for female and male samples are presented (Mann–Whitney *U* test)

An average of 36,414 ± 3,763 (mean ± *SD*) high quality, classifiable 16S rRNA gene sequences from the gut microbial communities of the Chinese concave‐eared frogs were obtained, with average counts per sample ranging from 35,719 ± 2,315 to 36,994 ± 999 (Mean ± *SD*). The sequences were classified into 2,289 OTUs based on 97% sequence identity. The gut microbial communities of both female and male frogs were dominated at the phylum level by Firmicutes (23.16% and 32.67%, respectively), Verrucomicrobia (24.24% and 26.23%), Bacteroidetes (27.25% and 11.61%), Proteobacteria (13.82% and 18.77%), and Fusobacteria (10.50% and 2.82%), and there was no significant difference in the relative abundance of the phyla and classes between sexes (*p* > 0.05 for all; Figure 1). The abundance of gut microbial communities in each individual at the phyla level was shown in Supporting Information Figure S2. All phylogenetic indices (Shannon index, Simpson’s index, Chao1 index, and the Good’s coverage index) confirmed that there was no significant difference in gut microbial diversity between males and females at the phylum and class levels (Mann–Whitney *U* test, *p* > 0.05 for all indices; Supporting Information Figure s3); however, inter‐sex differences were identified at lower taxonomic levels. For example, at the order level, significantly more reads were assigned to Enterobacteriales in female samples (10.12%) than in male samples (2.69%). At the family level, the relative abundance of Enterobacteriaceae was significantly higher in females than in males, whereas the opposite was observed for Rikenellaceae. Additionally, several microbial families also exhibited marked differences between sexes (Table 1).

**Table 1:** Differences in taxonomic composition of the intestinal microbiota of male vs. female Chinese concave‐eared frogs

Rank	Classification	Female (%)	Male (%)	*p*‐Value
Order	Enterobacteriales	10.120	2.691	0.022
Family	Enterobacteriaceae	10.120	2.691	0.022
Family	Peptostreptococcaceae	1.005	0.037	0.008
Family	Rikenellaceae	0.017	0.305	0.008
Genus	Unclassified of Erysipelotrichaceae	0.253	1.507	0.036
Genus	*Robinsoniella*	0.997	0.117	0.008
Genus	*Erysipelatoclostridium*	0.223	0.075	0.075
Genus	*Alistipes*	0.011	0.235	0.034

Note

Significant differences in microbial composition (relative abundance, %) at the genus, family, and order levels between male and female Chinese concave‐eared frogs are indicated. *p*‐values < 0.05 indicate significance, as calculated using a Mann–Whitney *U* test.

### 3.3 Functional analysis of metagenomic datasets

Metagenomic data analysis confirmed most of the dominant microbial phyla as determined by 16S rRNA sequencing, except for Fusobacteria, with Bacteroidetes, Firmicutes, Verrucomicrobia, and Proteobacteria identified as the four most dominant phyla (Supporting Information Figure S4). Among the 5,349,836 annotated genes and modules in the metagenomic dataset, 47.47% were identified as glycoside hydrolases (GH), 18.96% were assigned to carbohydrate‐binding module (CBM) families, 18.84% were glycosyltransferases (GT), and 10.41% belonged to carbohydrate esterase (CE) families. The GH catalytic modules contained 2,539,430 sequences belonging to 93 GH families, while the CBM modules included 1,014,146 sequences from 60 families. Enzymes related to chitin degradation are shown in Table 2. Phylogenetic analysis of these contigs indicated that the dominant phyla of chitin‐degrading bacteria were Bacteroidetes (39.53%), Firmicutes (37.50%), and Proteobacteria (17.18%). Specifically, Bacteroidia (38.63%), Clostridia (29.46%), and gammaproteobacteria (14.08%) appeared to be the main classes of chitin‐decomposing bacteria in the frog intestine (Supporting Information Table S4).

**Table 2:** Glycoside hydrolase (GH) and carbohydrate‐binding module (CBM) profiles of intestinal microbiota in Chinese concave‐eared frogs in relation to chitin degradation. Data are presented as the sum number of genes encoding the corresponding enzyme

	RTF	RTM	Total	Known chitin degradation activities
Chitinases				
GH18	109	57	163	Chitinase; lysozyme; endo‐β‐*N*‐acetylglucosaminidase; peptidoglycan hydrolase with endo‐β‐*N*‐acetylglucosaminidase; others
GH19	8	16	24	Chitinase; lysozyme
GH23	137	116	253	Lysozyme type G; peptidoglycan lyase; chitinase
Chitosanase				
GH7	0	1	1	Endo‐β‐1,4‐glucanase; reducing end‐acting cellobiohydrolase; chitosanase; endo‐β‐1,3‐1,4‐glucanase
GH8	12	4	16	Chitosanase; cellulase; licheninase; endo‐1,4‐β‐xylanase; reducing‐end xylose‐releasing exo‐oligoxylanase
GH46	8	1	9	Chitosanase
GH75	3	1	4	Chitosanase
GH80	1	0	1	Chitosanase
Lysozyme				
GH18	106	57	163	Chitinase; lysozyme; endo‐β‐*N*‐acetylglucosaminidase; peptidoglycan hydrolase with endo‐β‐*N*‐acetylglucosaminidase; others
GH19	8	16	24	Chitinase; lysozyme
GH22	0	2	2	Lysozyme type C; lysozyme type i; α‐lactalbumin
GH24	31	39	70	Lysozyme
Cellulases				
GH5	57	27	84	Endo‐β‐1,4‐glucanase/cellulase; endo‐β‐1,4‐xylanase; β‐glucosidase; β‐mannosidase; others
GH8	12	4	16	Chitosanase; cellulase; licheninase; endo‐1,4‐β‐xylanase; reducing‐end‐xylose releasing exo‐oligoxylanase
*N*‐acetylglucosaminidase				
GH18	106	57	163	Chitinase; lysozyme; endo‐β‐*N*‐acetylglucosaminidase; peptidoglycan hydrolase with endo‐β‐*N*‐acetylglucosaminidase; others
GH20	209	91	300	β‐hexosaminidase; lacto‐*N*‐biosidase; β‐1,6‐*N*‐acetylglucosaminidase; β‐6‐SO3‐*N*‐acetylglucosaminidase
GH73	98	58	156	Lysozyme; mannosyl‐glycoprotein endo‐β‐*N*‐acetylglucosaminidase; peptidoglycan hydrolase with endo‐β‐*N*‐acetylglucosaminidase specificity
GH84	43	23	66	*N*‐acetyl β‐glucosaminidase; hyaluronidase; [protein]‐3‐O‐(GlcNAc)‐l‐Ser/Thr β‐*N*‐acetylglucosaminidase
GH85	10	4	14	Endo‐β‐*N*‐acetylglucosaminidase
GH89	38	22	60	α‐*N*‐acetylglucosaminidase
GH111	1	0	1	Keratan sulfate hydrolase (endo‐β‐*N*‐acetylglucosaminidase)
GH116	16	13	29	β‐glucosidase; β‐xylosidase; acid β‐glucosidase/β‐glucosylceramidase; β‐*N*‐acetylglucosaminidase
Chitin‐binding function				
CBM2	2	10	12	Several of these modules have been shown to also bind chitin or xylan; others
CBM3	3	0	3	In one instance binding to chitin has been reported; others
CBM5	983	19	992	Chitin‐binding described in several cases; others
CBM12	23	17	40	The majority of these modules is found among chitinases where the function is chitin‐binding; others
CBM14	3	2	5	The chitin‐binding function has been demonstrated in several cases; others
CBM18	0	1	1	The chitin‐binding function has been demonstrated in many cases. These modules are found attached to a number of chitinase catalytic domains, but also in non‐catalytic proteins either in isolation or as multiple repeats; others
CBM19	0	2	2	Modules of 60–70 residues with chitin‐binding function
CBM73	5	1	6	Modules of approx 65 residues found on various enzymes active of chitin. Chitin‐binding function demonstrated for the *Cellvibrio japonicus* CjLPMO10A protein

The distribution of the genome among the general functional categories was assessed based on BLAST matches against the COG database. When the metagenomic data were included, the following categories were identified: carbohydrate transport and metabolism [G], amino acid transport and metabolism [E], inorganic ion transport and metabolism [P], energy production and conversion [C], coenzyme transport and metabolism [H], nucleotide transport and metabolism [F], and lipid transport and metabolism [I] (Supporting Information Figure s5). Some genes were categorized as “unknown function” (30.09% for female and 27.73% for male).

### 3.4 Comparison of gut microbiota between healthy and nematode‐infected individuals

The diversity of the gut bacterial communities of the nematode‐infected frogs was not significantly different from that of the healthy frogs, as confirmed by Shannon index, Simpson’s index, Chao1 index, and the Good’s coverage index analyses (Mann–Whitney *U* test, *p* > 0.05 for all indices).

**Figure 2:**
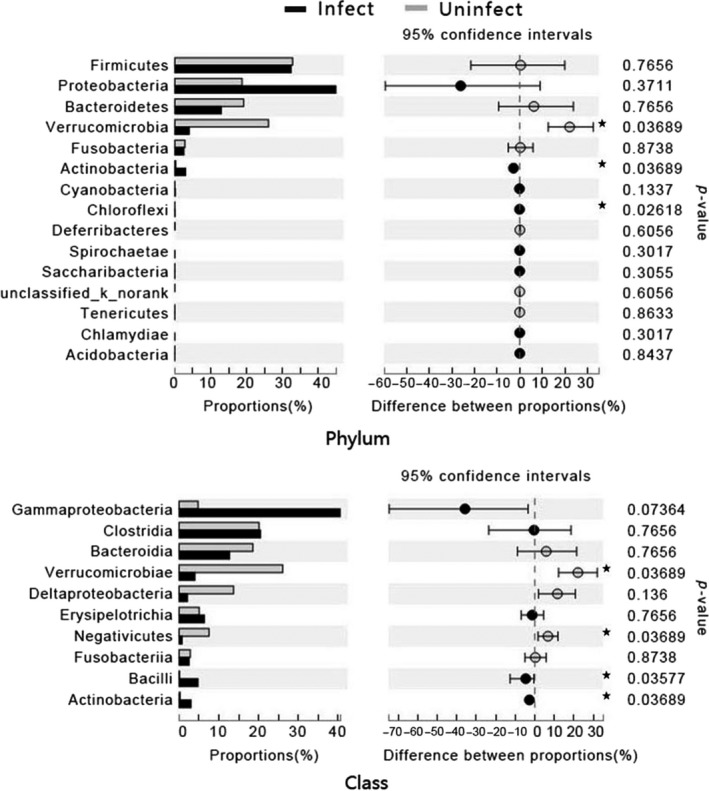
Comparison of the taxonomic compositions of the gut microbiota of the nematode‐infected and uninfected Chinese concave‐eared frogs. The relative abundances (percentage) of the microbiota at the phylum and class levels are presented. Asterisks indicate significant differences (Mann–Whitney *U* test: *p ≤ 0.05)

Investigation of the nematode‐infected frog samples showed that frog intestinal microbial communities exhibited a significant reduction in the relative abundance of Verrucomicrobia, Verrucomicrobiae, and Negativicutes, and a significant increase in the relative abundance of Bacilli and Actinobacteria at the phylum and class levels (Figure 2).

**Figure 3:**
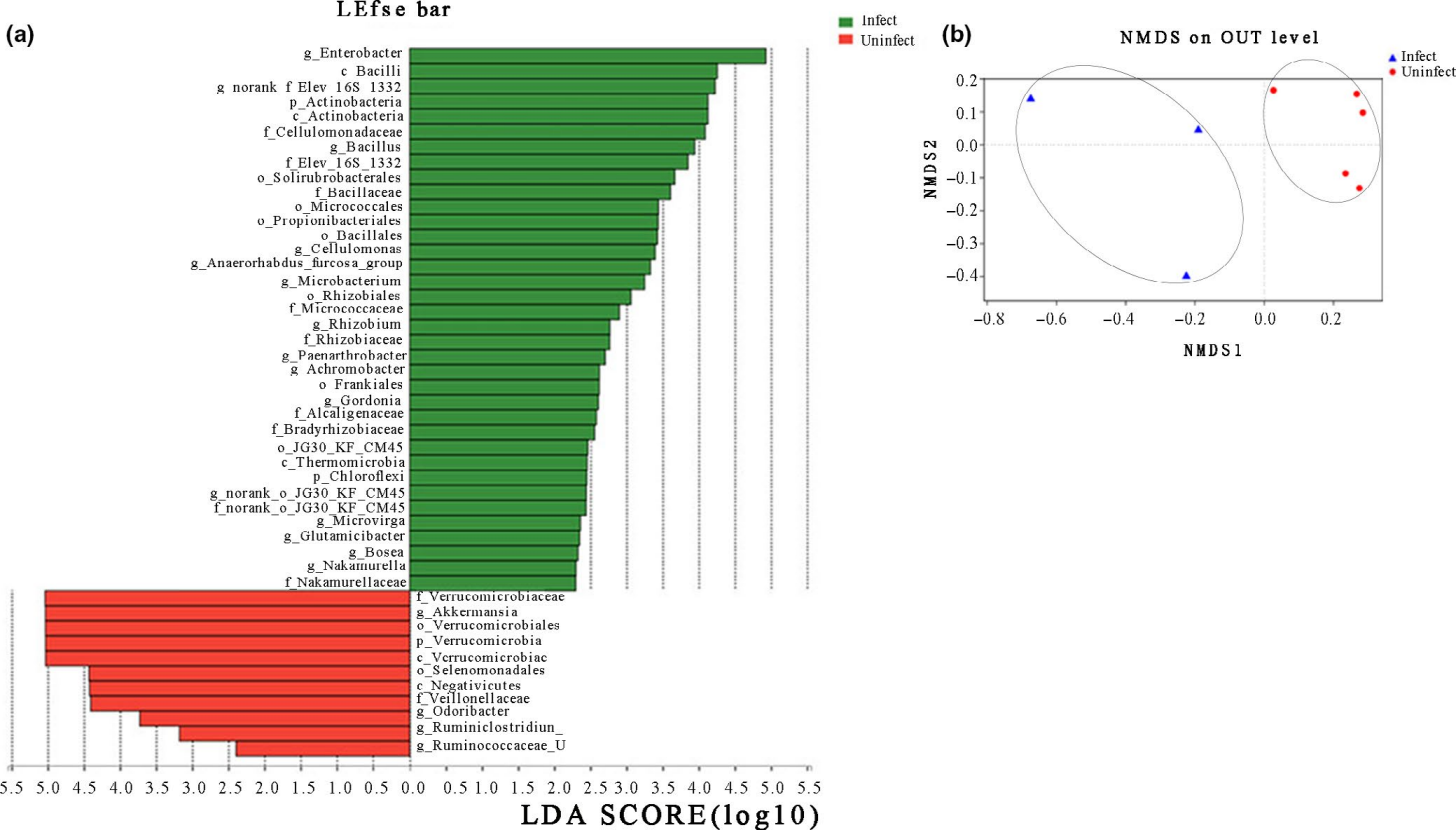
(a) Bacterial taxa that were differentially abundant in the gut microbiota profiles of nematode‐infected and uninfected Chinese concave‐eared frogs visualized using a log LDA score above 2.00. (b) NMDS analysis showing differences in gut microbiota between nematode‐infected and uninfected Chinese concave‐eared frogs

A supervised comparison using LEfSe was then performed to statistically define (at a log LDA threshold of 2.0) the differences in microbial composition between healthy and nematode‐infected frogs. This also confirmed that bacteria from the family Verrucomicrobiaceae, the genus Akkermansia, the order Verrucomicrobiales, and the class Verrucomicrobiae were more abundant in healthy individuals, while those from the genus Enterobacter, the class Bacilli, and the phylum Actinobacteria were more abundant in the infected individuals (Figure 3a). A clear distinction in the gut bacterial community structures of infected and uninfected frogs was also revealed by ANOSIM (*R* = 0.4154, *p* = 0.043) and NMDS analyses (Figure 3b).

## 4 DISCUSSION

There is a significant degree of variation in the dominant members of the gut microbial communities of vertebrates. For example, the communities of teleost fish are rich in Proteobacteria (Sullam et al., 2012), while tetrapod communities are dominated by Firmicutes and Bacteroidetes (Costello et al., 2012; Kohl & Yahn, 2016). In the present study, in addition to Firmicutes and Bacteroidetes, the phyla Verrucomicrobia and Proteobacteria were also abundant in the Chinese concave‐eared frog, as confirmed by both 16S rRNA sequencing and metagenomic analysis. Similar results have previously been obtained in other frog species (Huang et al., 2018; Vences et al., 2016), suggesting that Firmicutes, Bacteroidetes, Verrucomicrobia, and Proteobacteria are the dominant phyla in amphibian gastrointestinal tracts. These results are also consistent with the previous suggestion that animals housed in similar environments and with similar predation conditions tend to harbor similar microbial groups at the higher taxonomic levels (Kovacs et al., 2011).

Diet category or host trophic level (carnivorous, omnivorous, and herbivorous) is the major factor driving the composition and metabolism of gut microbiota (Han et al., 2016; Ley et al., 2008). Sexual dimorphism is a common phenomenon in amphibians (Shine, 1979). Former researches have showed that frogs with large body size tend to hunt for larger preys, while the small ones might tend to prey on smaller foods (Hirai, 2002; Houston, 1973; Lima & Moreira, 1993; Toft, 1980). The Chinese concave‐eared frog has a larger and smaller body sizes for females and males respectively, hence the types of food available to both sexes may slightly vary within the same region. The current study showed lack of similarity in the microbial diversity and relative abundance of communities between male and female concave‐eared frogs at the phylum and class levels based on 16S rRNA sequence data. However, significant differences in the gut microbial composition between sexes were observed at some of the lower taxonomic levels. The difference in composition of intestinal microbes at low levels may have resulted from weak differences in predation between sexes. Based on our current findings, we could also not determine whether these differences were caused by hormone–microbe interactions, sex‐specific immune responses, or other factors (Bolnick, Snowberg, Hirsch, & Lauber, 2014; Markle et al., 2014).

Insects, which constitute the staple diet of frogs, are rich in protein and chitin. Studies indicate that chitin degradation depends on several specific enzymes (Beier & Bertilsson, 2013). Goodrich and Morita (1977) found a direct correlation between the chitin content of the natural diet and bacterial chitinase activity in the stomach of marine piscivorous fish species. Microbial chitinases can degrade chitin into its monomeric or oligomeric components, thereby degrading the major component of the insect outer skeleton (Suganthi, Senthilkumar, Arvinth, & Chandrashekara, 2017). Chitinolytic enzymes in the digestive systems of marine fishes are derived from both the prey and enteric bacteria (Gutowska, Drazen, & Robison, 2004), while chitinases in the frog gut can be produced in the stomach (Fujimoto et al., 2004). However, to date, little is known about the chitinolytic activity of bacteria in the frog gut (Delsuc et al., 2014; Vences et al., 2016). The present study indicates that bacterial members of the frog gut microbiota can digest chitin using chitin‐degrading enzymes, shown by the presence of genes assigned to GH families and CAZy modules. Furthermore, the COG functional category profiles from the frog intestinal metagenomes showed an abundance of sequences associated with carbohydrate transport and metabolism, as well as many chitinolytic enzymes associated with Bacteroides. A large proportion of the proteins produced by Bacteroides species are used to break down polysaccharides and metabolize sugars (Xu, 2003). These enzymes play a fundamental role in the processing of complex molecules into simpler forms in the host intestine. The ability to harvest alternative energy sources from food might allow Bacteroides species to be more competitive than other bacteria in the frog intestine. Therefore, intestinal microbes may be a complementary pathway for frog digestion of chitin.

Parasitic nematodes, known as helminths, cause a wide range of diseases in humans and animals, and it is estimated that >10% of the world’s population is at risk of helminth infection every year (Crompton, 1999). The intestinal microbiota composition may reflect the state of the immune system and health of the host species (Round & Mazmanian, 2009). However, Lukeš, Stensvold, Jirků‐Pomajbíková, and Wegener Parfrey (2015) promoted the idea of some parasites being beneficial to the host rather than culprits of disease. For example, a mutualistic relationship exists between bullfrog tadpoles (*Rana catesbeiana*) and a tadpole‐specific gastrointestinal nematode (*Gyrinicola batrachiensis*; Pryor & Bjorndal, 2010). As yet, the complex interactions between helminths, gut microbiota, and the host have not been adequately studied in wild species (Kreisinger, Bastien, Hauffe, Marchesi, & Perkins, 2015). Therefore, in the current study, we examined the association between nematode infection and gut microbiota diversity and composition in wild concave‐eared frogs. We found that while nematode infection was not associated with changes in the overall gut microbiota diversity, there did appear to be an effect on the microbial community composition. This result is consistent with findings in wild mice, where helminth infection did not affect the diversity of the gut microbiota (Kreisinger et al., 2015). In addition, the gut microbial communities of the nematode‐infected and healthy frogs in the current study were clearly separated by ANOSIM (*R* = 0.5827, *p = *0.002) and NMDS analyses. Interestingly, the infected frogs seemed to exhibit higher inter‐individual variation, especially in terms of community structure. These results may demonstrate that nematode infection can increase heterogeneity of microbial communities among individuals.

The relative abundance of symbionts and pathogenic microbes also reflects the health status of the host species (Sekirov, Russell, Antunes, & Finlay, 2010). We found that the relative abundance of Verrucomicrobia was markedly reduced in nematode‐infected frogs compared with healthy frogs. Additionally, the relative abundance of some bacteria, such as Actinobacteria and Bacilli, increased in the infected frogs compared with the uninfected group. Actinobacteria have been associated with disease in humans (Abusleme et al., 2013), while Bacilli are highly abundant in the guts of several animal species and may enhance digestion by complementing the digestive enzymes in the gut, thereby improving nutrition through the provision of vitamins and amino acids to the host (Gandotra, Kumar, Naga, & Bhuyan, 2018; Voirol, Frago, Kaltenpoth, Hilker, & Fatouros, 2018). Therefore, our findings indicate that nematode infection of vulnerable Chinese concave‐eared frogs has complex implications for the gut microbiota, including loss of some beneficial microbes and increases in the abundance of some disease‐associated microbial taxa. As the functions of these bacteria in the concave‐eared frog have not been adequately described owing to the relatively low number of samples in this study, more work is needed to fill the gaps in our understanding of the interaction between helminths and the gut microbiota of this vulnerable species.

## CONFLICTS OF INTEREST

The authors declare that there are no competing interests.

## AUTHORS CONTRIBUTION

Hailong Wu, Bangding Xiao, Yilin Shu, and Pei Hong designed experiments. Dong Tang, Yilin Shu, Hui Qing, Huan Wang, and Oscar Omondi Donde collected samples and carried out experiments. Pei Hong and Yilin Shu analyzed experimental results and wrote the manuscript.

## ETHICS STATEMENT

All samples used in this study were collected with the permission of the Management Bureau of the Banqiao Provincial Natural Reserve. The animal experiments were performed under an animal ethics approval granted by Anhui Normal University.

## DATA ACCESSIBILITY

The 16S rRNA gene sequences and metagenome sequences from the frog gut microbiota samples reported in this study have been submitted to the NCBI Sequence Read Archive under accession numbers SRP131550 (https://www.ncbi.nlm.nih. gov/sra/SRP131550) and SRP130863 (https://www.ncbi.nlm.nih.gov/sra/SRP130863), respectively.
