# Supplementary figures and images for: Comparison of intestinal microbes in female and male Chinese concave‐eared frogs (Odorrana tormota) and effect of nematode infection on gut bacterial communities
Source: Microbiologyopen. 2018 Oct 11;8(6):e00749. doi: 10.1002/mbo3.749 (PMC6562124; doi:10.1002/mbo3.749)

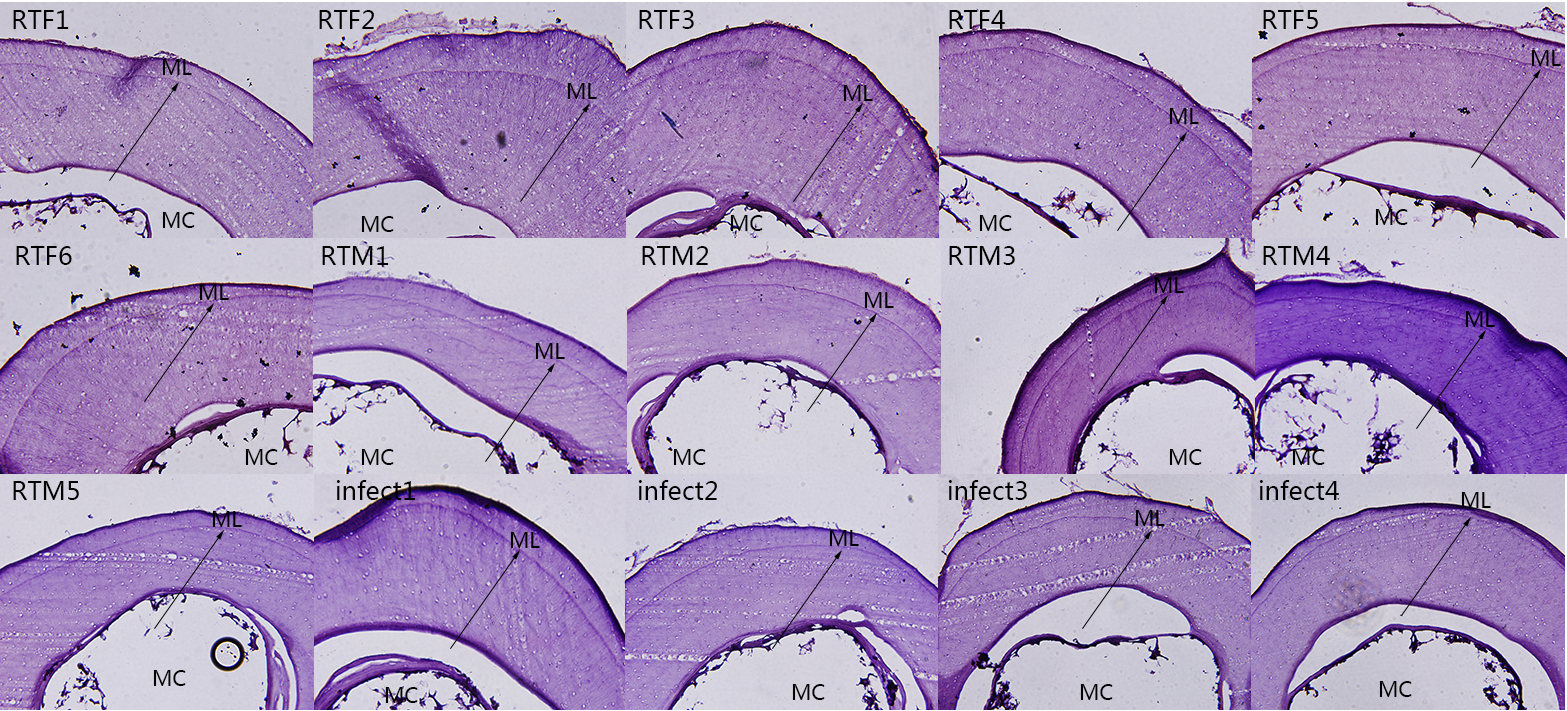

Supplement: Supplementary file 1 [file MBO3-8-e00749-s001.tif]

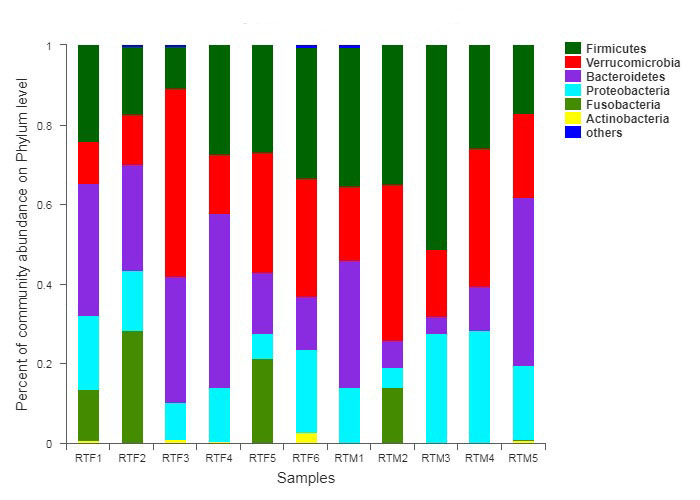

Supplement: Supplementary file 2 [file MBO3-8-e00749-s002.tif]

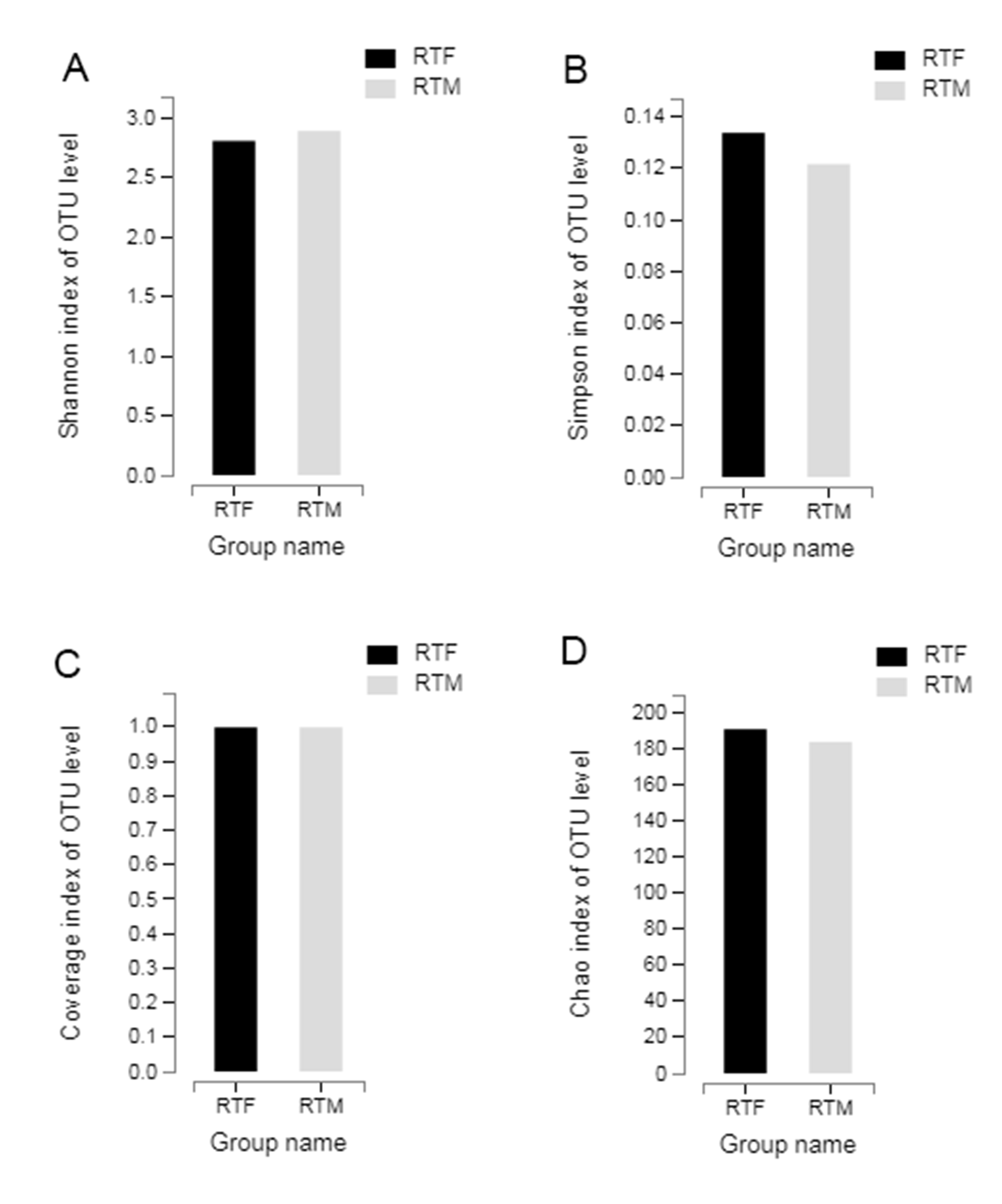

Supplement: Supplementary file 3 [file MBO3-8-e00749-s003.tif]

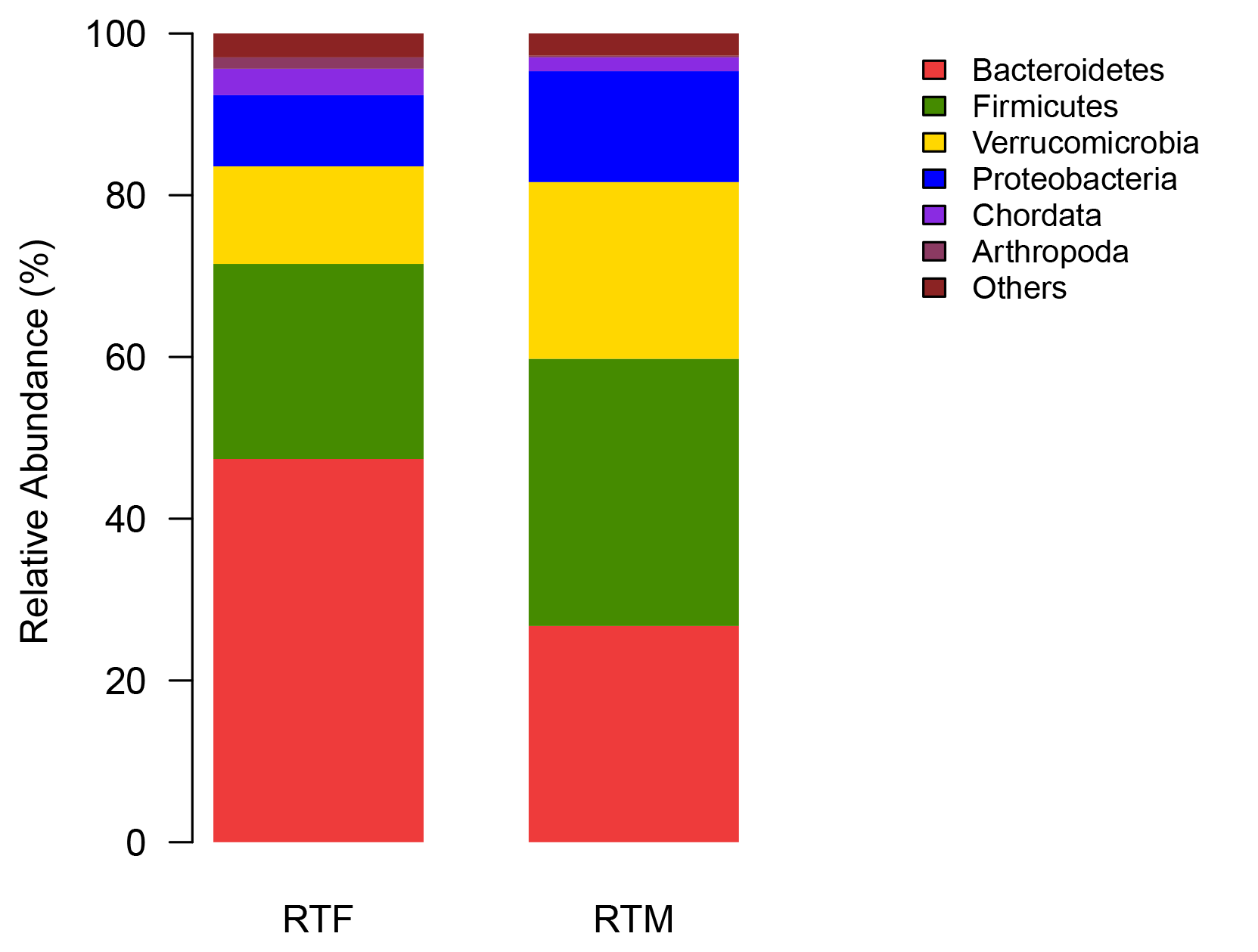

Supplement: Supplementary file 4 [file MBO3-8-e00749-s004.tif]

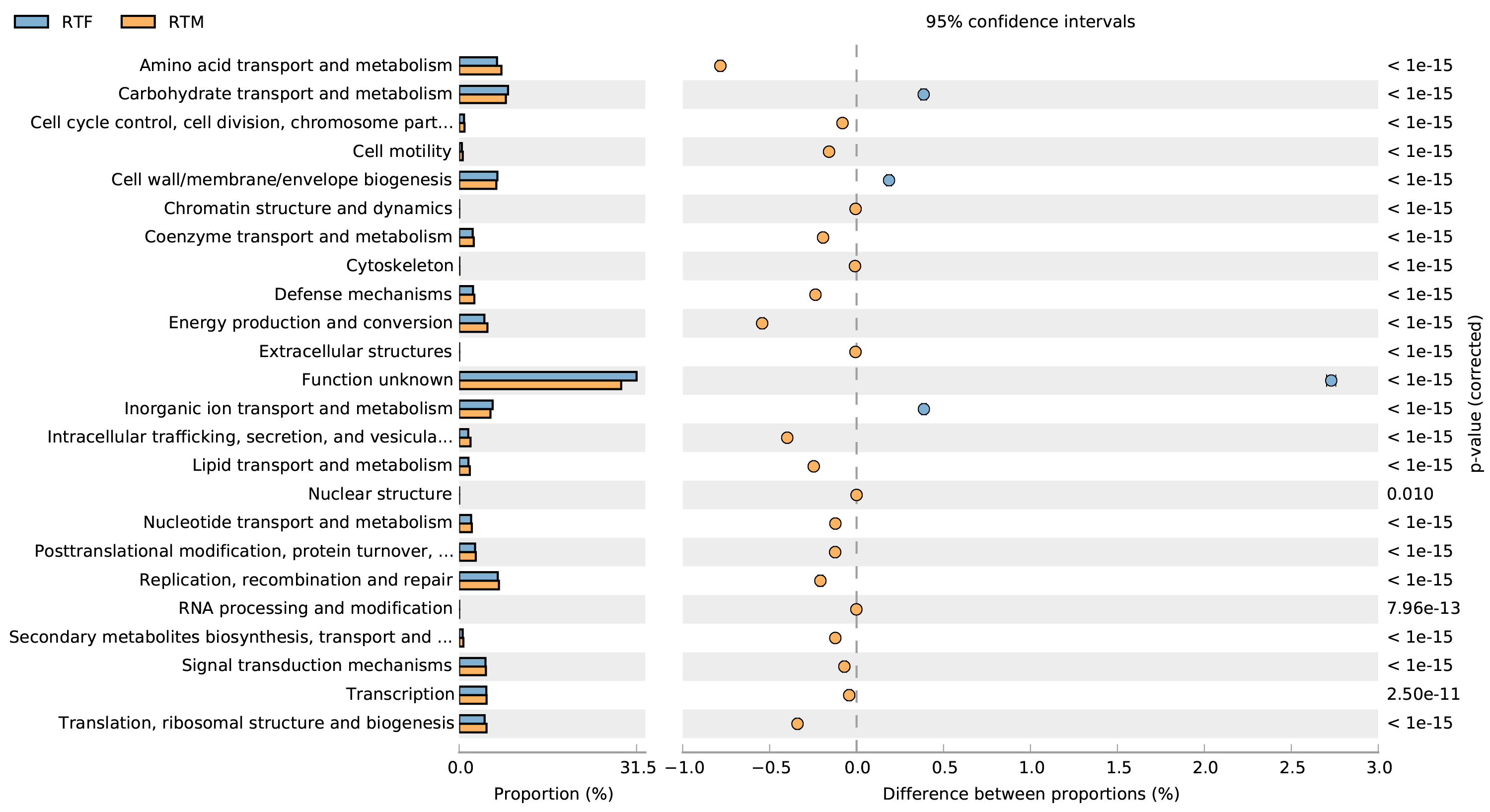

Supplement: Supplementary file 5 [file MBO3-8-e00749-s005.tif]
